# DELAYED HEADING DATE1 interacts with OsHAP5C/D, delays flowering time and enhances yield in rice

**DOI:** 10.1111/pbi.12996

**Published:** 2018-09-04

**Authors:** Huan Zhang, Shanshan Zhu, Tianzhen Liu, Chunming Wang, Zhijun Cheng, Xin Zhang, Liping Chen, Peike Sheng, Maohong Cai, Chaonan Li, Jiachang Wang, Zhe Zhang, Juntao Chai, Liang Zhou, Cailin Lei, Xiuping Guo, Jiulin Wang, Jie Wang, Ling Jiang, Chuanyin Wu, Jianmin Wan

**Affiliations:** ^1^ National Key Laboratory for Crop Genetics and Germplasm Enhancement Nanjing Agricultural University Nanjing China; ^2^ National Key Facility for Crop Gene Resources and Genetic Improvement Institute of Crop Science Chinese Academy of Agricultural Sciences Beijing China

**Keywords:** rice, heading date, GRAS, *Ehd1*, OsHAP5C/D, yield

## Abstract

Heading date is an important agronomic trait affecting crop yield. The GRAS protein family is a plant‐specific super family extensively involved in plant growth and signal transduction. However, GRAS proteins are rarely reported have a role in regulating rice heading date. Here, we report a GRAS protein DHD1 (Delayed Heading Date1) delays heading and enhances yield in rice. Biochemical assays showed DHD1 physically interacts with OsHAP5C/D both *in vitro* and *in vivo*. DHD1 and OsHAP5C/D located in the nucleus and showed that rhythmic expression. Both DHD1 and OsHAP5C/D affect heading date by regulating expression of *Ehd1*. We propose that DHD1 interacts with OsHAP5C/D to delay heading date by inhibiting expression of *Ehd1*.

## Introduction

Rice is one of the most important food crops in the world. Heading date has a significant impact on agronomic traits associated with yield (Jung and Muller, 2009). Photoperiod is the most important environmental factor affecting flowering time in rice. Many flowering time‐related genes have been cloned in rice. *Heading date 3a* (*Hd3a*) and *RICE FLOWERING LOCUS T1* (*RFT1*) are florigen proteins in rice (Chardon and Damerval, 2005; Komiya *et al*., 2009; Tsuji *et al*., 2013). *Hd3a* is activated under SD and inhibited under LD, whereas *RFT1* expression is increased at later developmental stages to promote flowering under LD condition (Komiya *et al*., 2008, 2009). *Hd3a* and *RFT1* expression is activated by a rice‐specific B‐type response factor encoded by *Early heading date 1* (*Ehd1*) under both SD and LD (Doi *et al*., 2004). *Ehd1* is up‐regulated by some proteins such as Ehd2/OsINDETERMINATE 1 (OsID1)/Rice INDETERMINATE 1 (RID1) (Matsubara *et al*., 2008; Park *et al*., 2008; Wu *et al*., 2008), Ehd3 (Matsubara *et al*., 2011), Ehd4 (Gao *et al*., 2013), VERNALIZATION INSENSITIVE 3‐LIKE 2 (OsVIL2) (Wang *et al*., 2013), SET domain group protein 724 (SDG724) (Sun *et al*., 2012a), OsMADS50 (Ryu *et al*., 2009) and OsMADS51 (Kim *et al*., 2007). In contrast, multiple proteins also act as negative regulators of *Ehd1*, including Grain yield and heading date 7 (Ghd7) (Xue *et al*., 2008), LEC2 and FUSCA3‐Like 1 (OsLFL1) (Peng *et al*., 2007), CONSTANS‐Like 4 (OsCOL4) (Lee *et al*., 2010), OsCOL10 (Tan *et al*., 2016), OsCOL13 (Sheng *et al*., 2016), Date To Heading on chromosome 7 (DTH7) (Gao *et al*., 2014), DTH8 (Wei *et al*., 2010), Heading date 16 (Hd16)/Early Flowering 1 (EL1) (Dai and Xue, 2010; Hori *et al*., 2013), SUPERNUMERARY BRACT (SNB), OsINDETERMINATE SPIKELET 1 (OsIDS1) (Lee *et al*., 2014), OsMADS56 (Ryu *et al*., 2009), ABA responsive element binding factor 1 (OsABF1) (Zhang *et al*., 2016) and Heme Activator Protein like 1 (OsHAPL1) (Zhu *et al*., 2017). Heading date 1 (Hd1) also activates *Ehd1* expression under SD, but functions as a repressor of *Ehd1* through interaction with Ghd7 under LD (Nemoto *et al*., 2016).

The plant‐specific GRAS (GAI, RGA and SCR) protein family, contains five conserved motifs named LHRI, VHIID, LHRII, PFYRE and SAW in their C‐termini (Bolle, 2004; Sun *et al*., 2011). This family can be divided into 10 subfamilies according to several independent phylogenetic analyses based on the relatively conserved motifs in the N‐terminal domains (Sun *et al*., 2011). GRAS proteins have been extensively studied in plants and are known involved in gibberellin (GA) signaling, root development, auxin response, stress responses, phytochrome (Phy) signaling, brassinosteroid (BR) signaling and control of tillering (Bolle, 2004; Sun *et al*., 2011, 2012b). SLENDER RICE1 (SLR1) protein is a negative regulator of GA signaling and belongs to the DELLA subfamily of GRAS (Ikeda *et al*., 2001). Tillering in rice is controlled by MONOCULM 1 (MOC1), which belongs to the *Arabidopsis* LATERAL SUPPRESSOR (AtLAS) subfamily (Li *et al*., 2003). DWARF AND LOW‐TILLERING (DLT), the DLT subfamily of GRAS, plays positive roles in brassinosteroid signaling (Tong *et al*., 2009). Rice homologs of SHORTROOT (SHR) and SCARECROW (SCR) have an evolutionarily conserved mechanism that defines a single endodermal layer similar to *Arabidopsis thaliana* (Cui *et al*., 2007). Two *Arabidopsis* Phytochrome A signal transduction (AtPAT) subfamily members, *Chitin‐Inducible Gibberellin‐Responsive 1* (*CIGR1*) and *CIGR2*, are responsive to N‐acetyl‐chitooligosaccharide elicitor and GA (Day *et al*., 2004). Recently, *OsGRAS23* was reported to be involved in drought stress response by regulating expression of stress‐responsive genes (Xu *et al*., 2015). However, GRAS proteins have not been reported in connection with heading date in rice.

Here, we identified a new GRAS protein DHD1 that delays heading date by down‐regulating expression of *Ehd1*,* Hd3a* and *RFT1*, and also enhances yield. DHD1 physically interacts with OsHAP5C and OsHAP5D both *in vitro* and *in vivo*. *OsHAP5C*/*D* also delays heading date by suppressing *Ehd1*. Both DHD1 and OsHAP5C/D have rhythmic expression modes. Our findings reveal functional roles of DHD1 in rice flowering time and rice yield.

## Results

### *DHD1* delays heading date in rice

Approximately 1,685 cDNAs of transcription factors from rice were fused to the VP64 activation domain, driven by the maize (*Zea mays*) ubiquitin (*pUbi*) promoter (Zhao *et al*., 2015). By analyzing the phenotypes of 57 751 independent transgenic plants of a *Japonica* rice variety Kitaake, we observed 30 independent lines, containing a *VP64*‐*LOC_Os11 g47920* construct, showed a delayed heading compared to wild type (WT, Figure S1). And then, we focused on the new gene (*LOC_Os11 g47920*) with unknown function, namely *DHD1* (*Delayed Heading Date1*).

**Figure 1:**
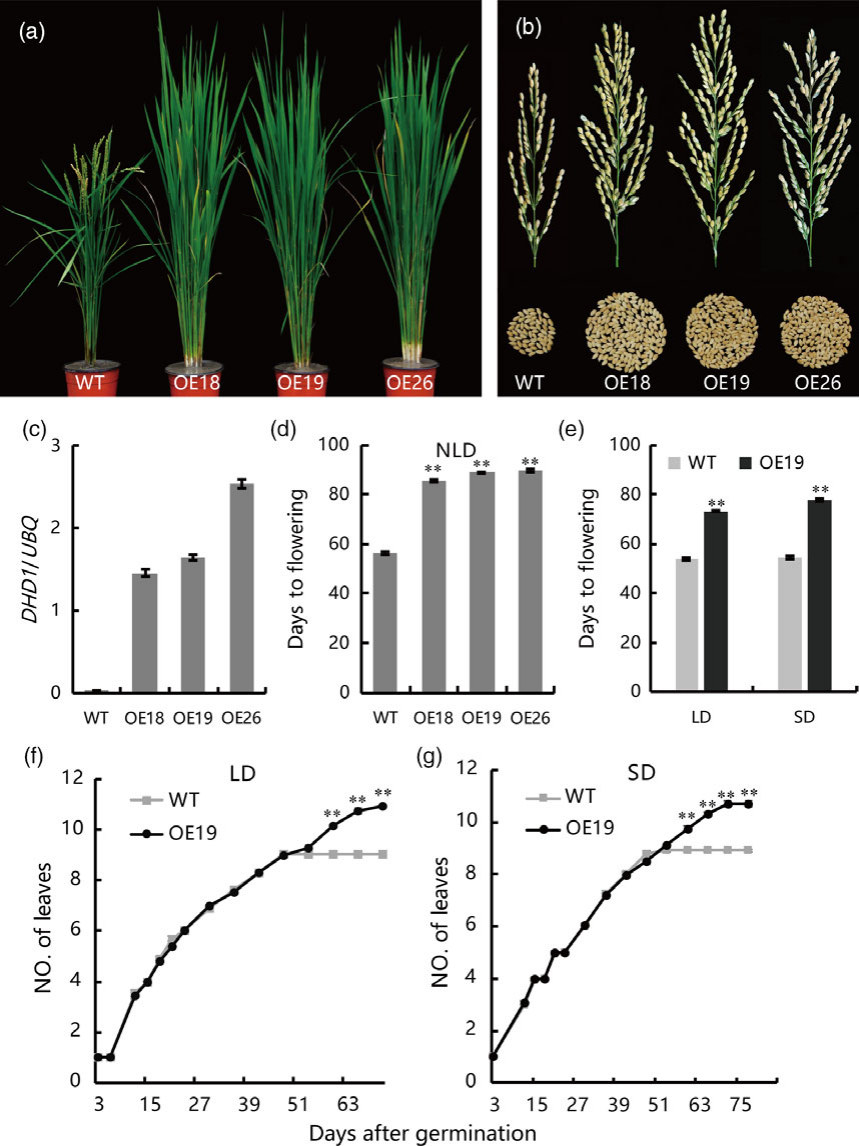
Phenotypes of *DHD1‐Flag* overexpression lines and wild type (WT) plants under natural LD, control LD and control SD conditions. (a) Phenotypes of overexpression and WT plants. Plants were grown under natural LD conditions for 70 days. OE18, OE19 and OE26, independent overexpression lines of *DHD1‐Flag*. (b) Main panicle size and grains per panicle of overexpression lines and WT. (c) Expression of *DHD1* in overexpression lines and WT. Means ± SE (*n *=* *3). (d) Heading dates of overexpression lines and WT. Means ± SE (*n *>* *15). (e) Flowering time of OE19 and WT under LD and SD conditions. Means ± SE (*n *>* *15). (f) Leaf emergence rate of OE19 and WT under LD conditions. Means ± SE (*n *>* *15). (g) Leaf emergence rate of OE19 and WT under SD conditions. Means ± SE (*n *>* *15), **P ≤* *0.01.

To eliminate potential effects of VP64 activation activities and further confirm the role of DHD1, we fused DHD1 to a 3 × Flag‐tag and overexpressed the fusion under control of *pUbi*. We obtained 13 independent transgenic lines, and found that all these independent transgenic lines had the delayed heading phenotype compared to WT. We randomly chose three independent transgenic lines (OE18, OE19 and OE26) and used their homozygous offsprings for further study. Under natural long day condition (NLD), flowering time of all three lines was delayed by about 1 month, and panicle size was significantly increased compared to WT (Figure 1a–d). Under controlled long day (LD, 14 h light/10 h darkness) and short day (SD, 10 h light/14 h darkness) conditions flowering time of OE19 plants was delayed by about 3 weeks relative to WT (Figure 1e). The leaf emergence rates of WT and OE19 were investigated until the heading stage. The leaf emergence rates were similar to WT, indicating that the delayed flowering time in the transgenic plants was not due to retarded growth (Figure 1f,g). Plant height, panicle length, primary branching and secondary branching of OE lines were significantly increased while tiller number and thousand grain weight were not changed (Table 1). These results suggest that *DHD1* may have potential to increase yield in breeding programs.

**Table 1:** Agronomic traits of wild type Kitaake and overexpression lines when planted in the field under NLD conditions

Trait	Kitaake	OE18	OE19	OE26
Plant height (cm)	65.66 ± 1.02	76.30 ± 0.84**	78.00 ± 1.07**	78.31 ± 0.98**
No. of tillers	19.00 ± 0.74	18.67 ± 1.15	16.80 ± 1.31	17.53 ± 0.94
Panicle length (cm)	13.03 ± 0.35	17.97 ± 0.22**	19.31 ± 0.61**	19.08 ± 0.43**
Primary branches, No./panicle	6.30 ± 0.15	16.40 ± 0.40**	14.90 ± 0.41**	16.30 ± 0.50**
Secondary branches, No./panicle	9.10 ± 0.53	24.40 ± 1.29**	26.60 ± 1.86**	26.90 ± 0.67**
Grains/panicle	58.40 ± 2.01	163.70 ± 6.16**	163.60 ± 7.16**	174.00 ± 2.51**
Thousand grain weight (g)	26.35 ± 0.12	25.23 ± 0.47	24.93 ± 0.64	24.92 ± 0.42

**P ≤* *0.01.

To further confirm the function of *DHD1* in plants, we created *DHD1* mutants by CRISPR‐*Cas9* technology in Kitaake, but there was no difference in flowering time between the mutants and Kitaake (Figure S2). Considering that Kitaake is an early flowering and photoperiod‐insensitive variety, we chose Nipponbare, a late flowering and photoperiod‐sensitivity variety, for further study to see if there is background effect. We created *DHD1*‐*RNAi* plants in Nipponbare background. There was no change in heading date when *DHD1*‐*RNAi* plants were planted in the field (Figure S5a–c). A genome‐wide search of rice revealed a homolog, DHD1L, with an amino acid sequence highly similar to DHD1 (Figure S3). We overexpressed *DHD1L* in Kitaake, and the heading date of the overexpression lines was also delayed (Figure S4). To eliminate the redundancy of *DHD1L*, we created a double mutant *dhd1 dhd1 l* in Nipponbare. We compared *DHD1*‐*RNAi*,* dhd1* and *dhd1 dhd1 l* phenotypes in the field, but found no significant difference in heading date compared to the WT (Figure S5). In addition, the agronomic traits such as plant height and panicle size of *dhd1* and *dhd1 dhd1 l* were decreased significantly (Table 2).

**Table 2:** Agronomic traits of wild type Nipponbare, *dhd1* and *dhd1 dhd1 l* when planted in the field under NLD conditions

Trait	Nip	*dhd1*	*dhd1 dhd1 l*
Plant height (cm)	100.54 ± 0.57	87.93 ± 0.58**	78.87 ± 0.82**
No. of tillers	16.70 ± 0.84	13.47 ± 0.39**	10.96 ± 0.41**
Panicle length (cm)	22.75 ± 0.24	20.12 ± 0.25**	19.37 ± 0.19**
Primary branches, No./panicle	13.11 ± 0.14	12.00 ± 0.21**	10.95 ± 0.26**
Secondary branches, No./panicle	30.28 ± 0.93	27.76 ± 0.72**	22.20 ± 0.71**
Grains/panicle	164.94 ± 3.10	151.52 ± 2.78**	121.60 ± 2.36**

**P ≤* *0.01.

### *DHD1* has a constitutive and rhythmic expression pattern and DHD1 is located in the nucleus

**Figure 2:**
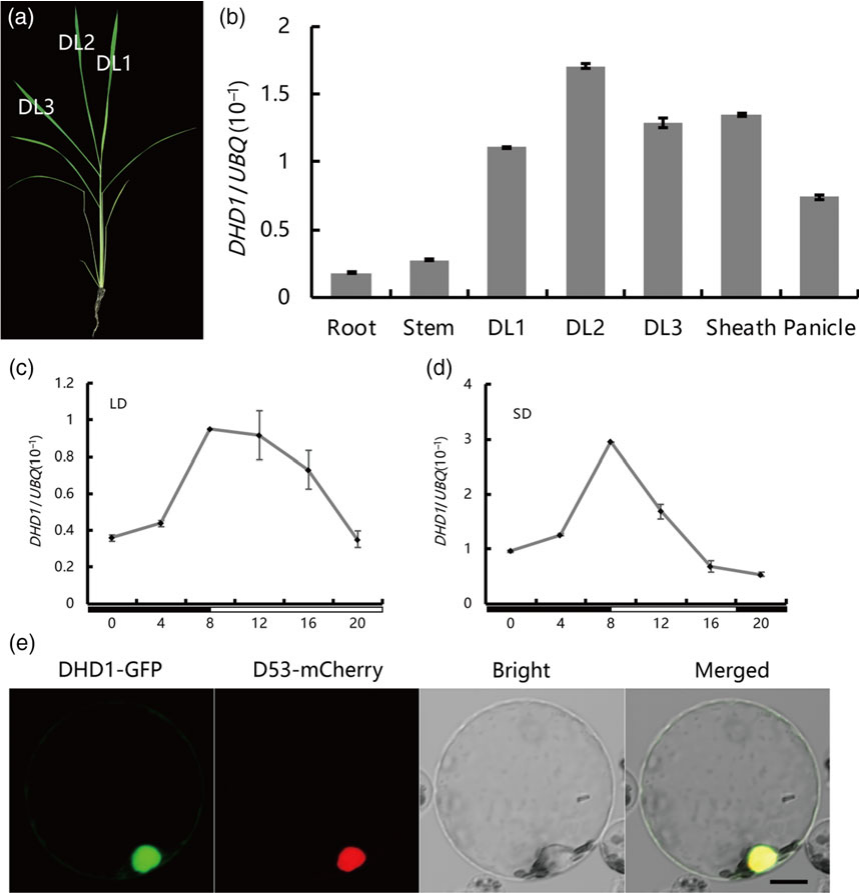
*DHD1* expression patterns, subcellular localization of protein and transcriptional activity. (a) Leaf samples at different developmental stages were obtained from 30 day old wild type plants. DL, developed leaf. (b) The relative expression levels of *DHD1* in different plant tissues. (c) Rhythmic expression pattern of *DHD1* under LD conditions. Black and white boxes denote dark and light periods respectively. (d) Rhythmic expression pattern of *DHD1* under SD conditions. Black and white boxes denote dark and light periods respectively. (e) Subcellular localization of DHD1‐GFP in rice protoplasts. Bar, 10 μm.

In order to examine the expression pattern of *DHD1*, quantitative real‐time PCR (qRT‐PCR) experiments were performed with different tissues (Figure 2a). Although *DHD1* was expressed in all tissues, expression levels were higher in leaves, leaf sheaths and panicles (Figure 2b). To investigate whether expression of *DHD1* was rhythmic, we assayed the expression every 4 h in a 24 h time course under LD and SD conditions. The transcription level of *DHD1* peaked at dawn and then slowly decreased until evening under both LD and SD conditions (Figure 2c,d). Our rice protoplast transient assays showed that DHD1‐GFP protein was mostly located in the nucleus, overlapping with the nuclear marker D53‐mCherry (Zhou *et al*., 2013) (Figure 2e).

### DHD1 interacts with OsHAP5C and OsHAP5D *in vitro* and *in vivo*

**Figure 3:**
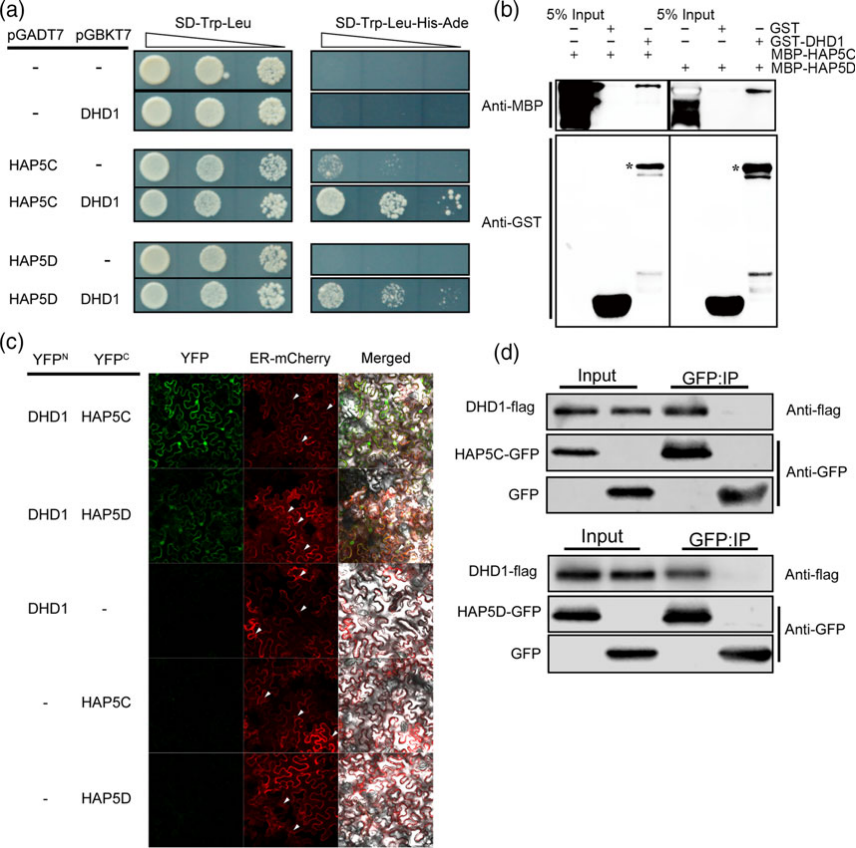
DHD1 interacts with OsHAP5C and OsHAP5D *in vitro* and *in vivo*. (a) Interactions of DHD1 with OsHAP5C/D in yeast strains. Triangle denotes 10‐fold dilution. (b) *In vitro* pull‐down analysis validates interactions of DHD1 with OsHAP5C/D. (c) BiFC analysis validates the interactions between DHD1 and OsHAP5C/D; the small triangles indicate the nucleus. (d) Co‐IP analysis in rice protoplasts verified interactions of DHD1 with OsHAP5C/D.

To investigate the action mode of DHD1, yeast two‐hybrid library screening with DHD1 as bait was performed and 73 positive clones were selected. By further confirmation of yeast‐two‐hybrid assay, we confirmed that OsHAP5C (LOC_Os03 g14669) and OsHAP5D (LOC_Os08 g38780) can interact with DHD1 in yeast (Figure 3a). A pull‐down assay was performed to confirm the physical interactions between DHD1 and OsHAP5C/D *in vitro* (Figure 3b).

BiFC and Co‐IP experiments were performed to further confirm the interactions *in vivo*. In the BiFC assay, YFP fluorescence was produced in tobacco leaves co‐infected by *Agrobacterium* containing DHD1‐YFP^N^ and OsHAP5C‐YFP^C^ or OsHAP5D‐YFP^C^. YFP fluorescence was not generated when the tobacco was co‐infected by DHD1‐YFP^N^ and empty YFP^C^. OsHAP5C‐YFP^C^ and OsHAP5D‐YFP^C^ with an empty YFP^N^ also did not result in YFP fluorescence (Figure 3c). The Co‐IP assay was performed in rice protoplasts. The vector containing *OsHAP5C‐GFP* or *OsHAP5D‐GFP* was co‐transformed with a vector containing *DHD1‐Flag* into rice protoplasts. Both OsHAP5C‐GFP and OsHAP5D‐GFP proteins co‐immunoprecipitated with the DHD1‐Flag protein, whereas GFP protein alone did not (Figure 3d). These results indicated that OsHAP5C and OsHAP5D could interact with DHD1 in tobacco and rice.

### OsHAP5C/D delayed heading date in rice

**Figure 4:**
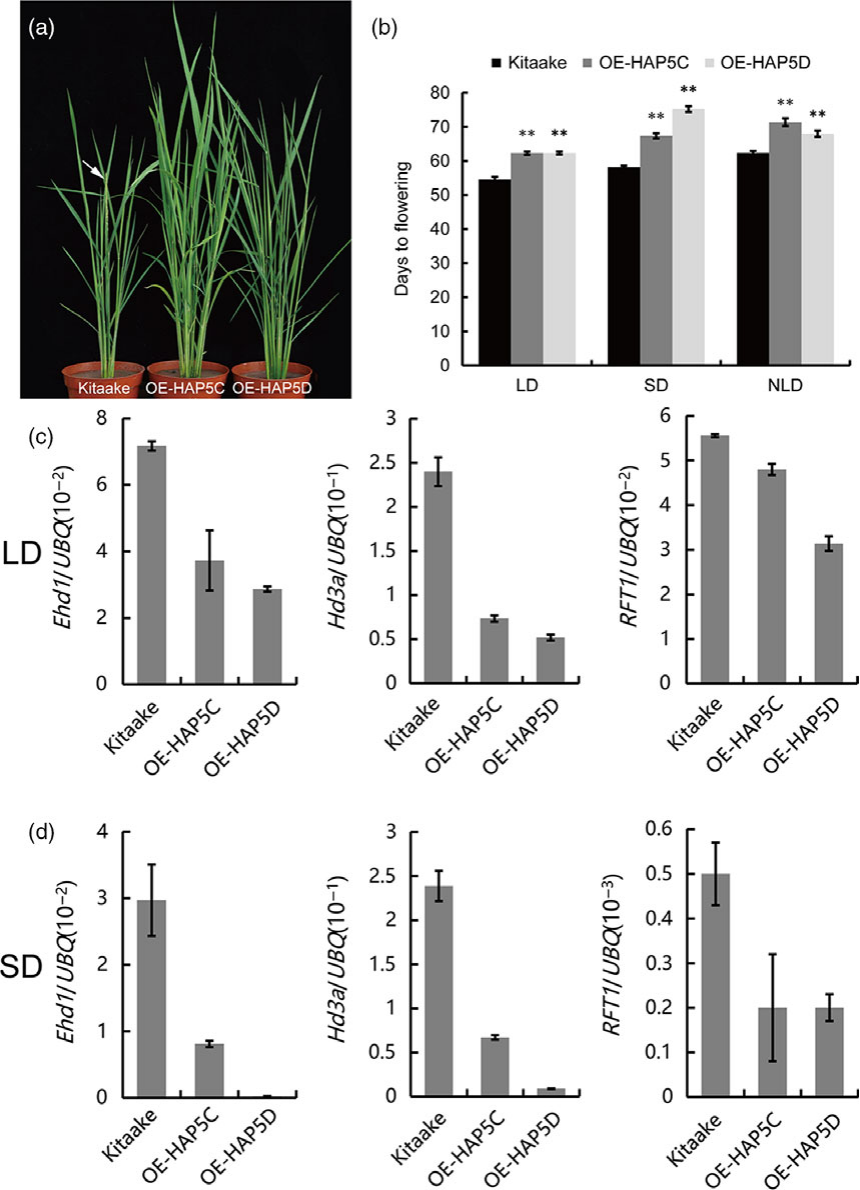
*OsHAP5C/D* overexpression delayed the heading date in rice. (a) Phenotypes of WT and *OsHAP5C/D* overexpression lines under NLD conditions. Arrow indicates the panicle. (b) Flowering time of WT and *OsHAP5C/D* overexpression lines under LD, SD and NLD conditions. Means ± SE (*n* > 12), **P ≤* *0.01. (c) Expression levels of *Ehd1*,* Hd3a* and *RFT1* in WT and *OsHAP5C/D* overexpression lines under LD conditions. Means ± SE. (d) Expression levels of *Ehd1*,* Hd3a* and *RFT1* in WT and *OsHAP5C/D* overexpression lines under SD conditions. Means ± SE.

By qRT‐PCR and subcellular localization experiments, we found that *OsHAP5C* and *OsHAP5D* were subject to rhythmic expression and the proteins were mainly located in the nucleus (Figure S6). To investigate whether *OsHAP5C*/*D* are involved in heading date, we created *OsHAP5C* and *OsHAP5D* overexpression plants. These plants showed later flowering than WT (Kitaake) under natural LD, control LD and control SD conditions (Figure 4a,b). qRT‐PCR showed that expression of *Ehd1*,* Hd3a* and *RFT1* was down‐regulated in *OsHAP5C*/*D* overexpressing plants (Figure 4c,d). These results indicated that *OsHAP5C*/*D* control heading date by regulating expression of *Ehd1*,* Hd3a* and *RFT1*.

### *DHD1* delays heading date by down‐regulating *Ehd1*,* Hd3a* and *RFT1*

**Figure 5:**
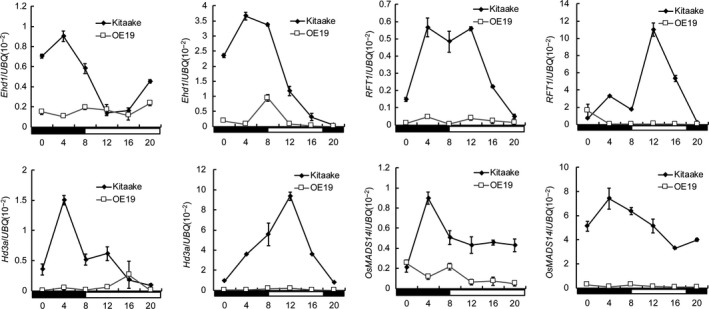
*DHD1* inhibited expression of downstream genes *Ehd1*,* Hd3a*,*RFT1* and *OsMADS14* under LD and SD conditions. Means ± SE. The black and white boxes denote dark and light periods, respectively.

In order to investigate downstream genes regulated by *DHD1*, we examined the expression of key genes involved in flowering in WT and OE‐DHD1‐19 under LD and SD conditions. qRT‐PCR data showed that expression of *Ehd1*,* Hd3a* and *RFT1* was greatly decreased under both LD and SD conditions in OE‐DHD1‐19 compared to WT, particularly, at the time of peak expression in WT. Expression of *OsMADS14*, a downstream gene of *Hd3a* and *RFT1*, was also significantly decreased (Figure 5). This suggests that *DHD1* delays flowering in rice by suppressing expression of *Ehd1*,* Hd3a* and *RFT1*.

We examined expression of some other heading‐related genes, including *OsPhyA*,* OsPhyB*,* OsPhyC*,* PHOTOPERIOD SENSITIVITY5* (*SE5*), *OsGIGANTEA* (*OsGI*), *OsMADS50*,* OsMADS51*,* OsMADS56*,* DTH2*,* DTH8*,* OsCOL4*,* RICE FLORICULA/LEAFY* (*RFL*), *Ehd2*,* Ehd3*,* Ehd4* and *Hd1*, but detected no significant changes (Figures S7 and S8). We also analyzed the expression of *DHD1* in mutants or near isogenic lines affecting heading date, in order to identify upstream genes that regulate *DHD1*. The results showed no changes of *DHD1* expression between WT and these mutants or near isogenic lines (Figures S9 and S10).

## Discussion

### DHD1 could be involved in GA and ABA‐related pathways of flowering

No change in the heading date of *DHD1*‐*RNAi*,* dhd1* or *dhd1 dhd1 l* plants when planted in the field. *DHD1* and *DHD1L* may be involved in other special rice flowering pathways. The homologous of DHD1, DELLA proteins, are involved in the GA flowering pathway in *Arabidopsis thaliana* (Wang *et al*., 2016). DELLA protein REPRESSOR of *ga1‐3* (RGA) can be degraded by GA (Willige *et al*., 2007) while ABA promotes its accumulation (Guo *et al*., 2014). Since DHD1 and DELLA proteins have conserved GRAS domains, we speculated that DHD1 has a similar function as DELLA. DHD1 was expressed transiently in the rice protoplasts, and treated with hormones GA, ABA and BR. We found that accumulation of DHD1 was increased under ABA treatment and decreased under GA treatment (Figure S11). Together, we speculated that DHD1 was involved in GA and ABA‐related pathways of flowering.

Salt stress delays flowering in *Arabidopsis*, which relies on the DELLA proteins that negatively regulate GA signaling and delay flowering by inhibiting downstream *CONSTANS* (*CO*) and *FLOWERING LOCUS T* (*FT*) expression (Achard *et al*., 2006; Li *et al*., 2007). Drought induces expression of *OsABF1*, which directly binds to the downstream promoter of *OsWARKY104* to activate its expression, which in turn, inhibits expression of *Ehd1* and delays flowering (Zhang *et al*., 2016). Previous studies have shown that the *Lilium longiflorum* SCR‐like (LISCL) subfamily proteins may be involved in the stress response, and recently, OsGRAS23, a member of the LISCL subfamily of rice, has been reported to be involved in drought response (Xu *et al*., 2015). We observed that the *dhd1 dhd1 l* double mutant flowered earlier than WT and the *dhd1* single mutant under drought and salt treatment. There was no significant difference in flowering time under normal conditions (Figure S12). We therefore hypothesized that DHD1 may be involved in the flowering regulation of stress, which remains to be further investigated.

### DHD1 interacts with OsHAP5C/D and participates in regulating heading date

Heme activator protein (HAP) proteins, also known as NUCLEAR FACTOR Y (NF‐Y) proteins, are a class of regulatory proteins widely found in fungi, animals and plants (Mantovani, 1999). In *Arabidopsis*, NF‐Y proteins bind *CCAAT* elements to form long‐distance chromatin loops on the *FT* promoter and initiate expression of *FT* by recruiting CO (Cao *et al*., 2014). The NF‐Y complex also partially modulates trimethylated H3K27 levels of the *SUPPRESOR OF OVEREXPRESSION OF CONSTANS 1* (*SOC1*) promoter by interacting with a H3K27 demethylase, RELATIVE OF EARLY FLOWERING 6 (REF6) (Hou *et al*., 2014). In rice, *DTH8*/*OsHAP3H* delays heading by decreasing the expression of *Ehd1* and *Hd3a* under LD (Wei *et al*., 2010). Li *et al*. (2016) overexpressed a series of HAP family genes and found that *OsHAP3D*,* OsHAP3E*,* OsHAP5A* and *OsHAP5B* delayed flowering under LD conditions. Furthermore, an OsHAPL1‐DTH8‐Hd1 complex was reported to function as a repressor of heading (Zhu *et al*., 2017). Yeast two‐hybrid library screening showed that DHD1 can interact with OsHAP5C and OsHAP5D. Furthermore, yeast two‐hybrid experiments and pull‐down experiments demonstrated that DHD1 physically interacts with OsHAP5C and OsHAP5D *in vitro* (Figure 3a,b). BiFC experiments in tobacco and the Co‐IP experiments in rice protoplasts verified these interactions *in vivo* (Figure 3c,d). Overexpression of *OsHAP5C*/*D* also delays heading in Kitaake under LD, SD and NLD conditions (Figure 4a,b), as well as reducing expression of *Ehd1*,* Hd3a* and *RFT1* (Figure 4c,d), in a similar manner to lines overexpressing *DHD1*. These results suggest that DHD1 and OsHAP5C/D may play roles in the same flowering pathway. We also created single and double mutants of *OsHAP5C* and *OsHAP5D*, but these had no effect on heading date relative to WT when grown in the field (Figure S13). Considering there are 11 HAP5 subunits in rice (Li *et al*., 2016), it is likely that many other *HAP5* genes play redundant roles.

### DHD1‐OsHAP5C/5D inhibits flowering by repressing *Ehd1* expression

It is generally considered that there are two flowering regulation pathways in rice: the *Hd1*‐mediated pathway and the *Ehd1*‐mediated pathway, which modulate expression of the florigen genes *Hd3a* and *RFT1* to control heading date (Sun *et al*., 2014; Tsuji *et al*., 2011). Additional genes directly regulate the expression of *Hd3a* and *RFT1*, such as *DTH2* (Wu *et al*., 2013), *OsCO3* (Kim *et al*., 2008) and *RFL* (Rao *et al*., 2008). It was reported that Hd1 interacts with Ghd7 and binds directly to a *cis*‐regulatory region in *Ehd1* to repress its expression (Nemoto *et al*., 2016). Drought, GA and low temperature also affect expression of *Ehd1* to control the flowering time (Cho *et al*., 2017). Thus, *Ehd1* may be an important integrator in regulating flowering time in rice. *DHD1* delayed heading by inhibiting *Ehd1* expression (Figure 5). NF‐YA, or HAP2, binds to *CCAAT* elements and recruits other HAP subunit proteins to bind the promoter (Gnesutta *et al*., 2017). There are several *CCAAT* elements in the promoter of *Ehd1*. We hypothesize that it is possible that other HAP2 proteins bind to the *Ehd1* promoter and recruit HAP5s and DHD1 to form a complex that regulates flowering. The relationships between DHD1 and HAP proteins and *Ehd1* remain to be further studied.

### DHD1 regulates heading date and has potential to increase yield in rice

The GRAS proteins are involved in many aspects of plant development and signal transduction in rice (Bolle, 2004; Sun *et al*., 2012b). However, the relationship between GRAS proteins and heading date is less reported. Here, we describe a GRAS family protein encoded by *DHD1* in rice. Overexpression of *DHD1* in Kitaake, a short‐season variety, delayed heading date and caused increases in a number of agronomic traits such as panicle length, primary branch number, secondary branch number and grain number per panicle, without a change number of tillers per plant. Thus, we speculated *DHD1* could be utilized to improve agronomic traits including yield in rice breeding.

## Experimental procedures

### Plant materials and growth conditions

The genotypes used for transformation were ssp. *japonica* varieties Kitaake and Nipponbare. All plants were grown in paddy fields at Beijing (116°13′E, 39°54′N) during summer, representing natural long day conditions. Plants were also grown in climate chambers with controlled LD (10 h darkness, 25 °C/14 h light, 30 °C) and SD (14 h darkness, 25 °C/10 h light, 30 °C) conditions with about 800 μmol/m^2^/s light intensity and 70% relative humidity.

### Vector construction and rice transformation

To generate *DHD1*,* DHD1L*,* OsHAP5C* and *OsHAP5D* overexpressing plants, full‐length CDSs of *DHD1*,* DHD1L*,* OsHAP5C* and *OsHAP5D* were amplified, and PCR products were subcloned into binary vector *pCAMBIA1390* using an In‐Fusion Advantage PCR Cloning Kit (Clontech, Beijing, China). To knockdown the *DHD1* gene, construct *pCUbi1390‐ΔFAD2* (*Ubi* promoter and a *FAD2* intron inserted into *pCAMBIA1390*) was used as an RNAi vector (Tan *et al*., 2014). Both antisense and sense versions of a specific 172 bp fragment from the coding region were amplified (primer pairs DHD1‐RNAi‐1 and DHD1‐RNAi‐2; Table S1), and inserted into *pCUbi1390‐ΔFAD2* to form the RNAi construct *pUbi‐dsRNAi‐DHD1*. To knockout the *DHD1* gene, 20 bp gene‐specific spacer sequence of the target gene (Table S1) was cloned into the entry vector *pOs‐sgRNA* and then subcloned into the destination vector containing the CAS9 expression cassette using the Gateway LR Clonase II Enzyme mix [Invitrogen, Shanghai, China; (Miao *et al*., 2013)]. To develop *dhd1 dhd1 l* double mutant, we constructed a *CRISPR‐Cas9* vector containing guide RNAs targeting *DHD1* and *DHD1L* respectively. Mutants of *hap5c*/*d* were created in the same way. The resulting plasmids were transformed into *Agrobacterium tumefaciens* strain EHA105 and then introduced into rice variety Kitaake or Nipponbare as described previously (Hiei *et al*., 1994). Primer sequences for construction of these vectors are listed in Table S1.

Total RNA was isolated from frozen tissues using a ZR Plant RNA MiniPrep Kit (ZYMO Research, Beijing, China) and reverse transcribed using a QuantiTect reverse transcription kit (Qiagen, Shanghai, China) according to the manufacturer's protocol. qRT‐PCR was performed with a SYBR premix Ex Taq Kit (TaKaRa, Dalian, China) according to the operation manual and amplified in an ABI 7500 using primers listed in Table S1. Data from three biological replicates were analyzed following the relative quantification method (Livak and Schmittgen, 2001). Primer sequences for qRT‐PCR are listed in Table S1.

### Phenotype measurement of agronomic traits

Various agronomic traits, including days to heading, plant height, tillers number, panicle length, primary branches number, secondary branches number, grain number per panicle and thousand grains weight (1000‐grain weight), were manually measured. Days to heading were counted from seed sowing to first panicle heading about 1–2 cm of each plant. The reproductive tillers having panicles with filled grains were counted for tillers number. Thousand grain weight was determined by measuring the weight of harvested seeds that were air‐dried in a glasshouse and oven‐dried at 50 °C until they reached ~14.0% moisture content. Panicle‐related traits containing panicle length, primary branches number, secondary branches number and grain number per panicle were measured from main tillers of 15 plants in WT and transgenic lines respectively (Kim *et al*., 2018). All data are analyzed by the *LSD*‐test.

### Subcellular localization

The full length cDNAs of *DHD1*,* OsHAP5C* and *OsHAP5D* were fused at the C‐terminal with GFP in the *pAN580* vector, and then transiently expressed the resulting construct in rice leaf protoplasts to explore the subcellular localization (Table S1). The method for rice leaf protoplast transformation is available in a previous report (Zhang *et al*., 2011). Fluorescence signals were detected by a ZESS LSM880 confocal microscope.

### Yeast two‐hybrid assay

The full length *DHD1* cDNA was amplified, inserted into the *pGBKT7* vector (Table S1), and then transformed into the yeast strain AH109. BD‐DHD1 was used for yeast two‐hybrid library screening experiment (Yeastmaker™ Yeast Transformation Manual User, Clontech). The complete coding sequences of the *OsHAP5C* and *OsHAP5D* genes were inserted into the *pGADT7* vector and co‐transformed into yeast with *pGBKT7‐DHD1* as described above (Table S1).

### *In vitro* pull‐down assay

The coding region of *DHD1* was inserted into the prokaryotic expression vector *pGEX4T‐1* for fusing the GST‐tag. The CDSs of *OsHAP5C* and *OsHAP5D* were inserted into *pMAL‐c2X* for fusing the MBP‐tag (Table S1). The vectors were transformed into the expression strain *Escherichia coli* BL21. Fusion proteins GST‐DHD1 and MBP‐OsHAP5C/D were induced with 0.5 mm IPTG at 16 °C for 12 h. Pull‐down assays were performed as previously reported (Miernyk and Thelen, 2008). MBP and GST antibodies with horseradish peroxidase (MBL) were used in western blotting experiments.

### Bimolecular fluorescence complementation (BiFC) analysis

The coding region of *DHD1* was inserted into the *pSPYNE173* vector and *OsHAP5C*/*D* were inserted into the *pSPYCE* (*M*) (Table S1). Fusion proteins DHD1‐YNE and OsHAP5C/D‐YCE were transiently expressed in 5‐6‐week‐old *Nicotiana benthamiana* leaves. The method for infiltrating *N*. *benthamiana* leaves with *Agrobacterium* strain EHA105 followed a previous report (Waadt and Kudla, 2008). Fluorescence signals were detected by a ZESS LSM880 confocal microscope 48–72 h after infiltration.

### *In vivo* co‐immunoprecipitation (Co‐IP) assay

The CDS of *DHD1* with a 3 × Flag‐tag was inserted into the *pAN580* vector. *OsHAP5C*/*D* were also inserted into *pAN580* vector for fusing GFP at the C‐terminal (Table S1). The vectors were co‐transformed into rice protoplasts as described above. The Co‐IP assay was performed as previously reported (Yang *et al*., 2014). Western blotting was detected by the Flag and GFP antibodies with horseradish peroxidase (MBL).

### Hormone treatment experiment

The *pAN580‐DHD1‐Flag* vector was transformed into rice protoplast as described above. The protoplasts were divided into four groups, three of which were separately treated with 10 μm GA, ABA and BR. Protein was extracted from each group after 4 h and subjected to western blotting.

## Supporting information


**Figure S1** VP64‐DHD1 overexpression plants flowered later than the wild type Kitaake.
**Figure S2** Heading date of *dhd1* mutant in Kitaake background under NLD, LD and SD conditions.
**Figure S3** Phylogenetic tree and protein sequence alignment of DHD1 and DHD1L
**Figure S4** *DHD1L* overexpression plants flowered later than the wild type Kitaake.
**Figure S5** Phenotypes of wild type Nipponbare, *DHD1‐RNAi*,* dhd1* and *dhd1 dhd1 l* plants grown in the field under NLD.
**Figure S6** Rhythmic expression pattern of *OsHAP5C*/*D* and subcellular localization of proteins.
**Figure S7** Relative expression levels of heading date‐related genes in wild type Kitaake and OE19 overexpression lines.
**Figure S8** Relative expression levels of heading date‐related genes in wild type Kitaake and OE19 overexpression lines.
**Figure S9** The relative expression levels of *DHD1* in heading date‐related mutants or near‐isogenic lines.
**Figure S10** The relative expression levels of *DHD1* in the heading date‐related mutants or near‐isogenic lines.
**Figure S11** Accumulation of DHD1‐Flag protein in rice protoplasts subjected to ABA, GA and BR hormone treatments.
**Figure S12** Flowering time of NIP, *dhd1* and *dhd1 dhd1 l* under normal, salt and drought conditions in SD climate chamber.
**Figure S13** Heading date of *hap5c*,* hap5d* and *hap5c hap5d* grown in the field under NLD condition.

**Table S1** Primers used in the current work.
