# Supplementary material for: DELAYED HEADING DATE1 interacts with OsHAP5C/D, delays flowering time and enhances yield in rice
Source: Plant Biotechnol J. 2018 Sep 4;17(2):531–9. doi: 10.1111/pbi.12996 (PMC6335081; doi:10.1111/pbi.12996)
Supplement: Supplementary file 1 — Figure S1 VP64‐DHD1 overexpression plants flowered later than the wild type Kitaake. Figure S2 Heading date of dhd1 mutant in Kitaake background under NLD, LD and SD conditions. Figure S3 Phylogenetic tree and protein sequence alignment of DHD1 and DHD1L Figure S4 DHD1L overexpression plants flowered later than the wild type Kitaake. Figure S5 Phenotypes of wild type Nipponbare, DHD1‐RNAi, dhd1 and dhd1 dhd1 l plants grown in the field under NLD. Figure S6 Rhythmic expression pattern of OsHAP5C/D and subcellular localization of proteins. Figure S7 Relative expression levels of heading date‐related genes in wild type Kitaake and OE19 overexpression lines. Figure S8 Relative expression levels of heading date‐related genes in wild type Kitaake and OE19 overexpression lines. Figure S9 The relative expression levels of DHD1 in heading date‐related mutants or near‐isogenic lines. Figure S10 The relative expression levels of DHD1 in the heading date‐related mutants or near‐isogenic lines. Figure S11 Accumulation of DHD1‐Flag protein in rice protoplasts subjected to ABA, GA and BR hormone treatments. Figure S12 Flowering time of NIP, dhd1 and dhd1 dhd1 l under normal, salt and drought conditions in SD climate chamber. Figure S13 Heading date of hap5c, hap5d and hap5c hap5d grown in the field under NLD condition. [file PBI-17-531-s002.docx]

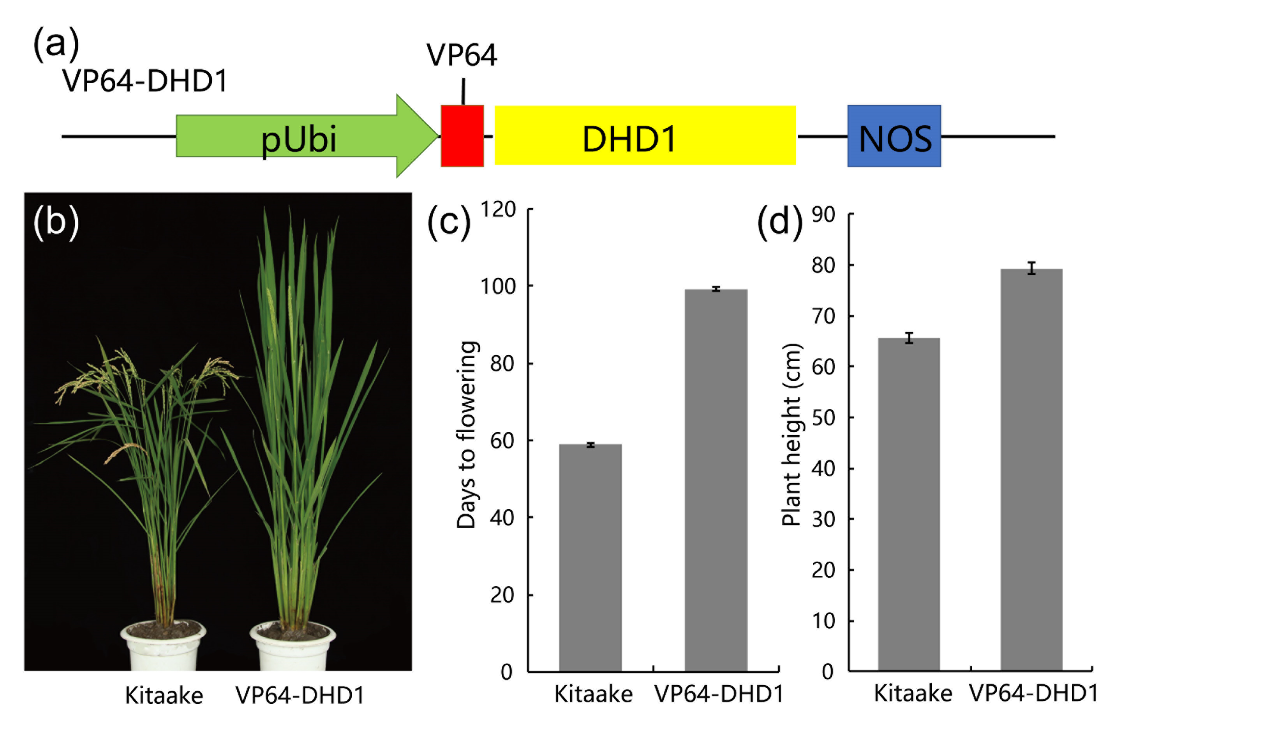


Figure S1. *VP64-DHD1* overexpression plants flowered later than the wild type Kitaake.

(a) Diagram of *VP64-DHD1* overexpression vector. (b) Phenotypes of wild type Kitaake and *VP64-DHD1* overexpression plants under NLD. (c) Flowering time of wild type Kitaake and *VP64-DHD1* overexpression plants under NLD. (d) Plant height of wild type Kitaake and *VP64- DHD1* overexpression plants under NLD.


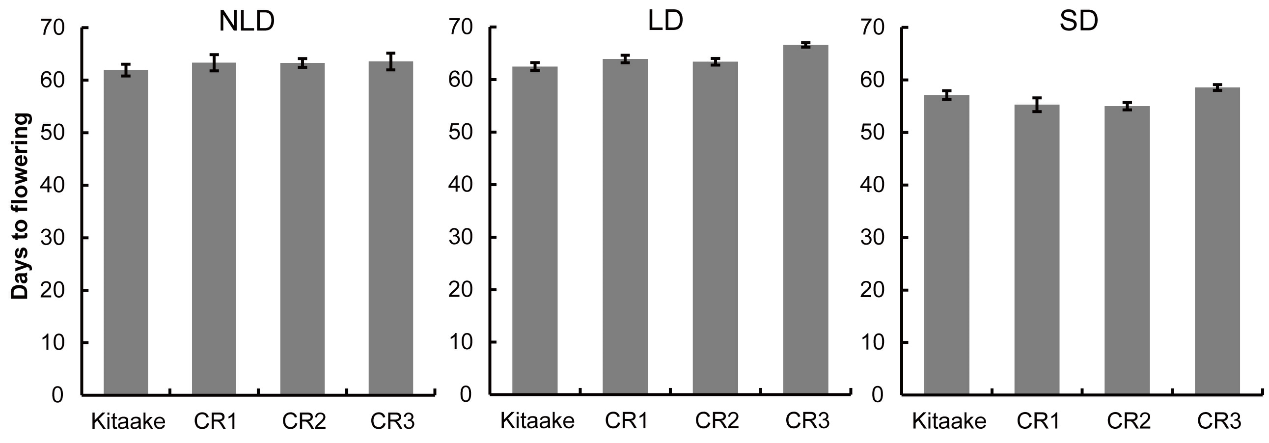


Figure S2. Heading date of *dhd1* mutant in Kitaake background under NLD, LD and SD conditions. CR1, CR2 and CR3 represent independent transgenic lines of *dhd1* mutant. Means ± SE (n>12).


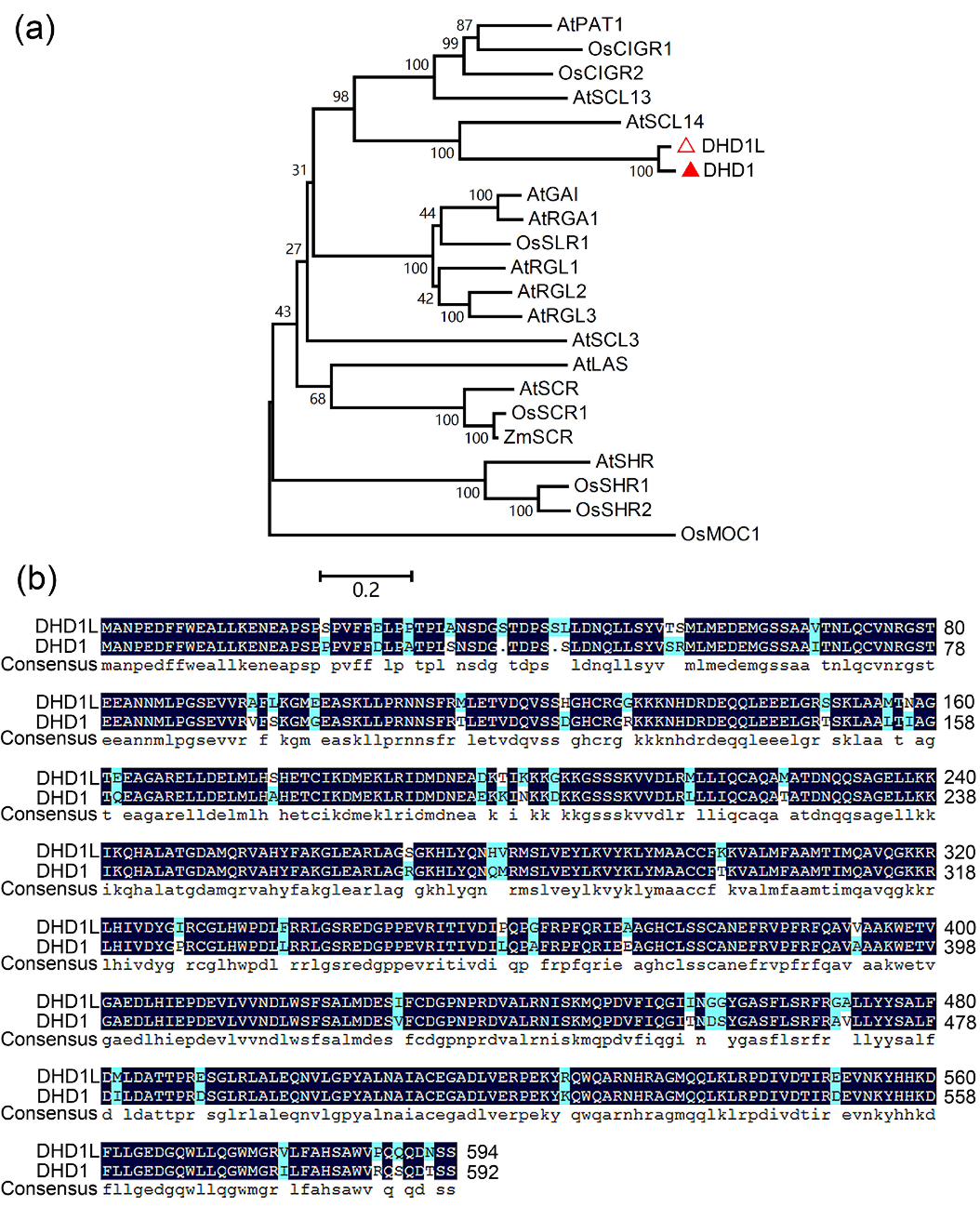


Figure S3. Phylogenetic tree and protein sequence alignment of DHD1 and DHD1L

(a) The tree was constructed using the neighbor-joining algorithm and a bootstrap of 1,000 replications. Solid red and hollow red triangles indicate the positions of DHD1 and DHD1L, respectively. The NCBI accessions of other proteins in the phylogenetic tree: AtPAT1, NP_199626.1; OsCIGR1, BAT02004.1; OsCIGR2, XP_015646745.1; AtSCL13, NP_193456.4; AtSCL14, NP_172233.1; AtGAI，NP_172945.1; AtRGA1, NP_178266.1; OsSLR1, BAS85987.1; AtRGL1, NP_176809.1; AtRGL2, NP_186995.1; AtRGL3, NP_197251.1; AtSCL3, NP_175459.1; AtLAS, NP_175954.1; AtSCR, NP_190990.1; OsSCR1, BAT12470.1; ZmSCR, NP_001168484.1; AtSHR, NP_195480.1; OsSHR1, BAT02401.1; OsSHR2, BAS84831.1; OsMOC1, BAS98577.1. (b) Protein sequence alignment of DHD1 and DHD1L.


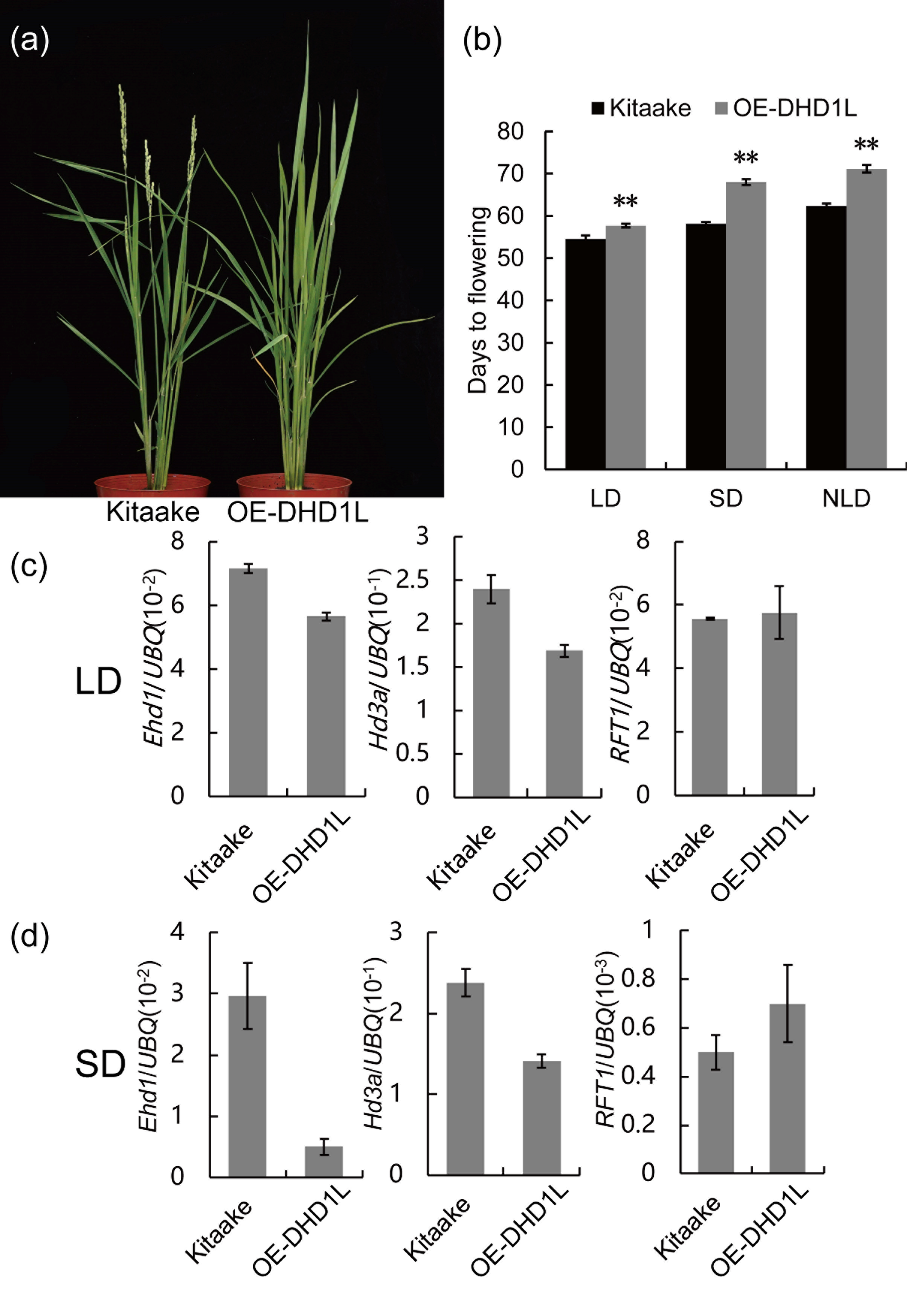


Figure S4. *DHD1L* overexpression plants flowered later than the wild type Kitaake.

(a) Phenotypes of wild type Kitaake and *DHD1L* overexpression plants under NLD. (b) Flowering times of wild type Kitaake and *DHD1L* overexpression plants under LD, SD and NLD. Means ± SE (*n*>12), ***p*≤0.01. (c) Transcriptional levels of *Ehd1*, *Hd3a* and *RFT1* in wild type and *DHD1L* overexpression plants under LD. Means ± SE, ***p*≤0.01. (d)Transcriptional levels of *Ehd1*, *Hd3a* and *RFT1* in wild type and *DHD1L* overexpression plants under SD. Means ± SE, ***p*≤0.01.





Figure S5. Phenotypes of wild type Nipponbare, *DHD1-RNAi*, *dhd1,* and *dhd1* *dhd1l* plants grown in the field under NLD.

(a) Phenotypes of wild type Nipponbare and *DHD1-RNAi* plants grown in the field under NLD conditions. Nip, wild type Nipponbare; *RNAi-1*, *RNAi-2* and *RNAi-3*, independent transgenic lines of *DHD1-RNAi*. (b) Expression levels of *DHD1* in wild type Nipponbare and *DHD1-RNAi* plants. Means ± SE. (c) Flowering time of wild type Nipponbare and *DHD1-RNAi* plants grown in the field under NLD conditions. Means ± SE (*n*>12). (d) Phenotypes of wild type Nipponbare, *dhd1* and *dhd1* *dhd1l* plants grown in the field under NLD. (e) Panicle size of wild type Nipponbare, *dhd1* and *dhd1* *dhd1l* plants grown in the field under NLD. (f) Flowering times of wild type Nipponbare, *dhd1* and *dhd1* *dhd1l* plants grown in the field under NLD conditions. Means ± SE (*n*>12).


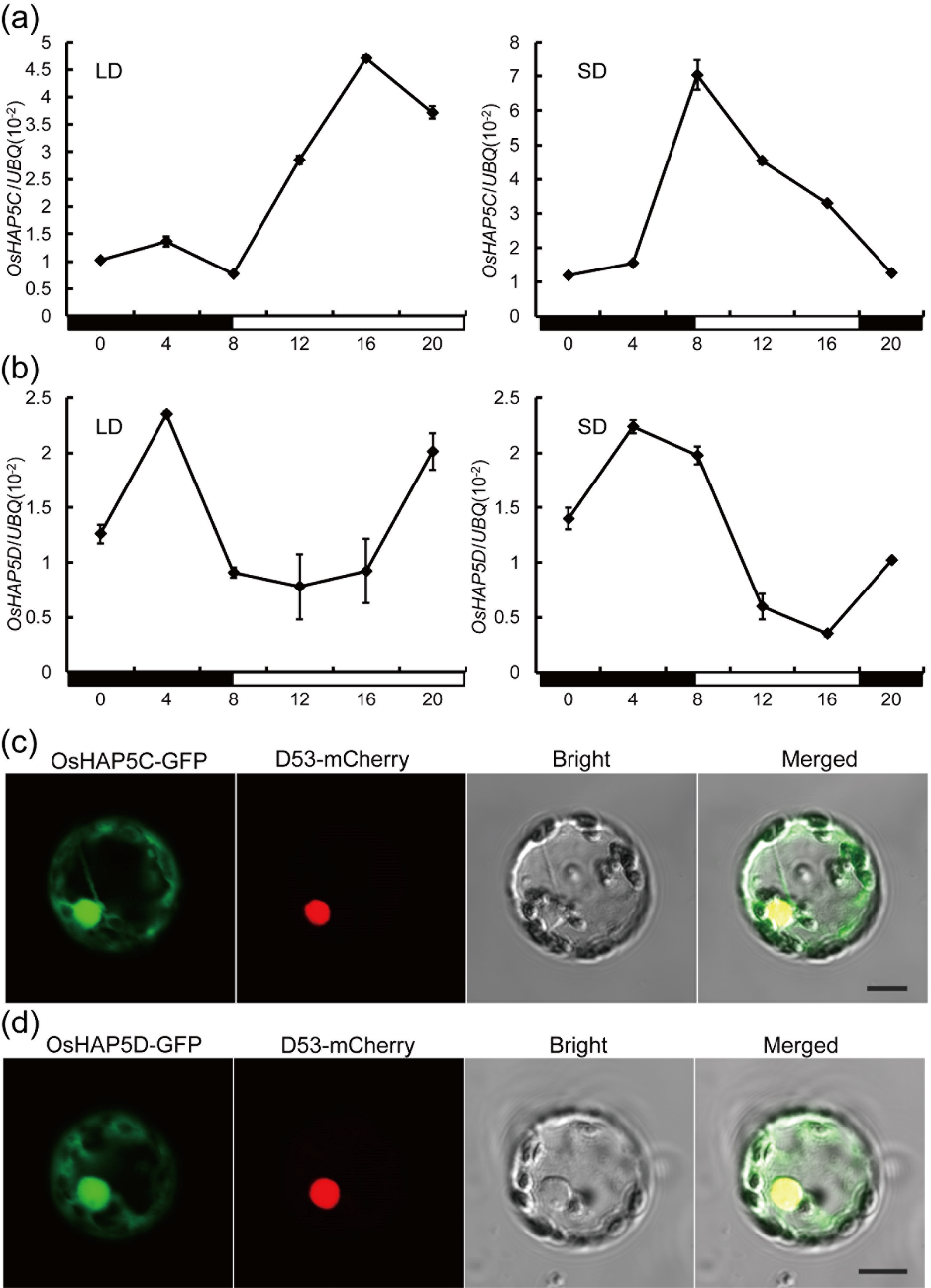


Figure S6. Rhythmic expression pattern of *OsHAP5C*/*D* and subcellular localization of proteins.

(a) Rhythmic expression pattern of *OsHAP5C* under LD and SD conditions. Means ± SE. Black and white boxes denote dark and light periods, respectively. (b) Rhythmic expression pattern of *OsHAP5D* under LD and SD conditions. Means ± SE. Black and white boxes denote dark and light periods, respectively. (c) Subcellular localization of OsHAP5C-GFP in rice protoplasts. Bar, 10 μm. (d) Subcellular localization of OsHAP5D-GFP in rice protoplasts. Bar, 10 μm.


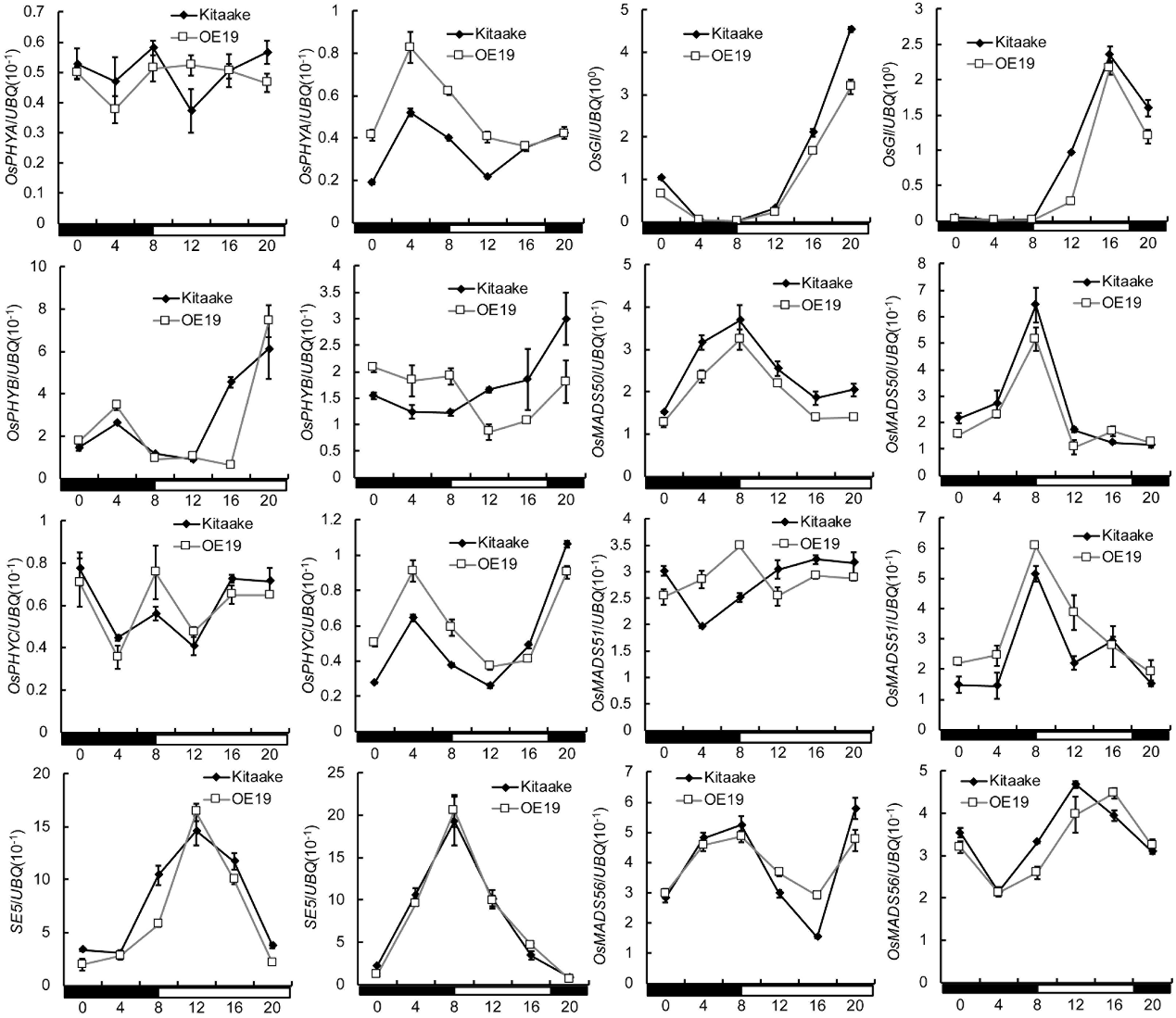


Figure S7. Relative expression levels of heading date-related genes in wild type Kitaake and OE19 overexpression lines. Means ± SE. Black and white boxes denote dark and light periods, respectively.


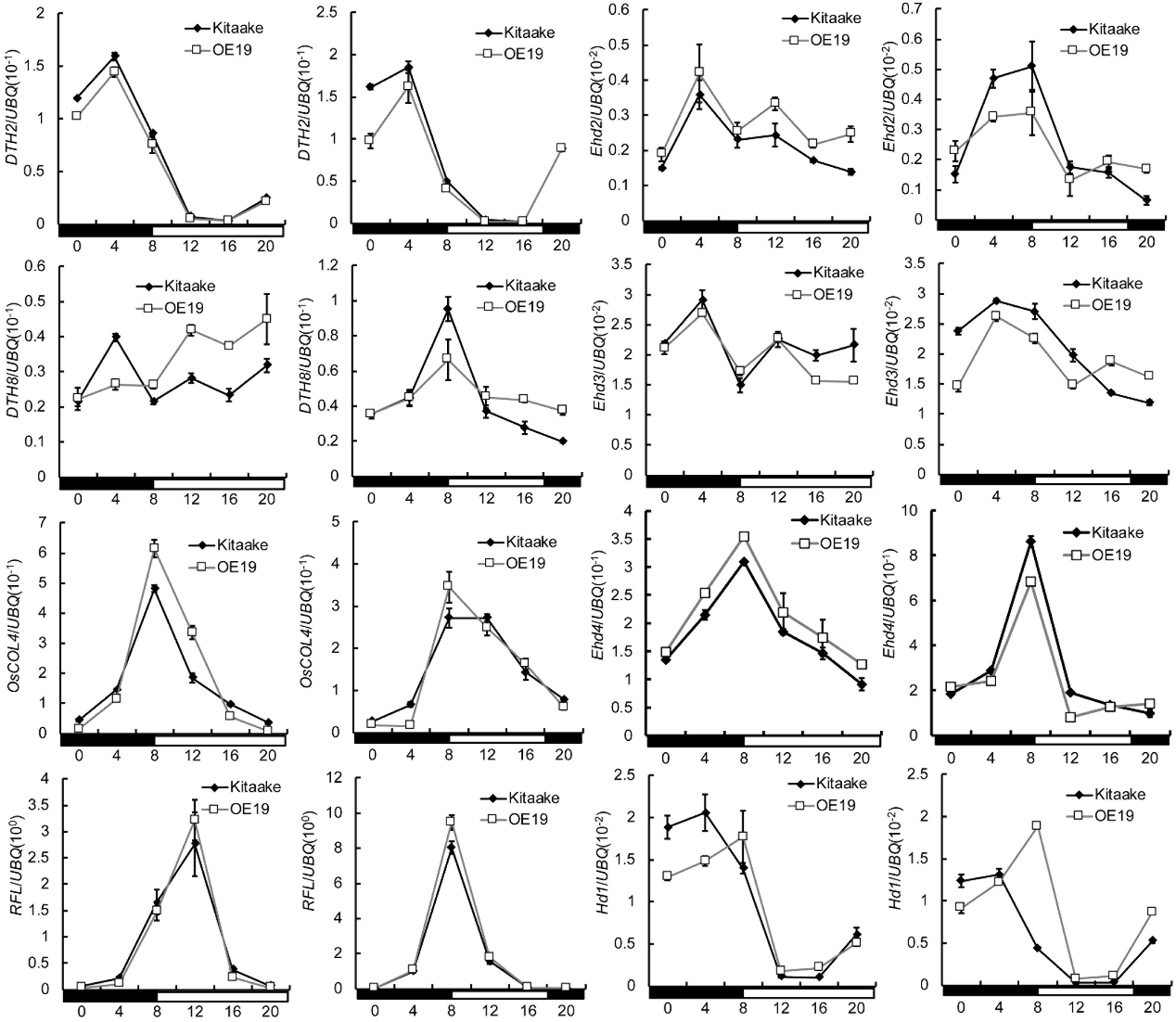


Figure S8. Relative expression levels of heading date-related genes in wild type Kitaake and OE19 overexpression lines. Means ± SE. Black and white boxes denote dark and light periods, respectively.


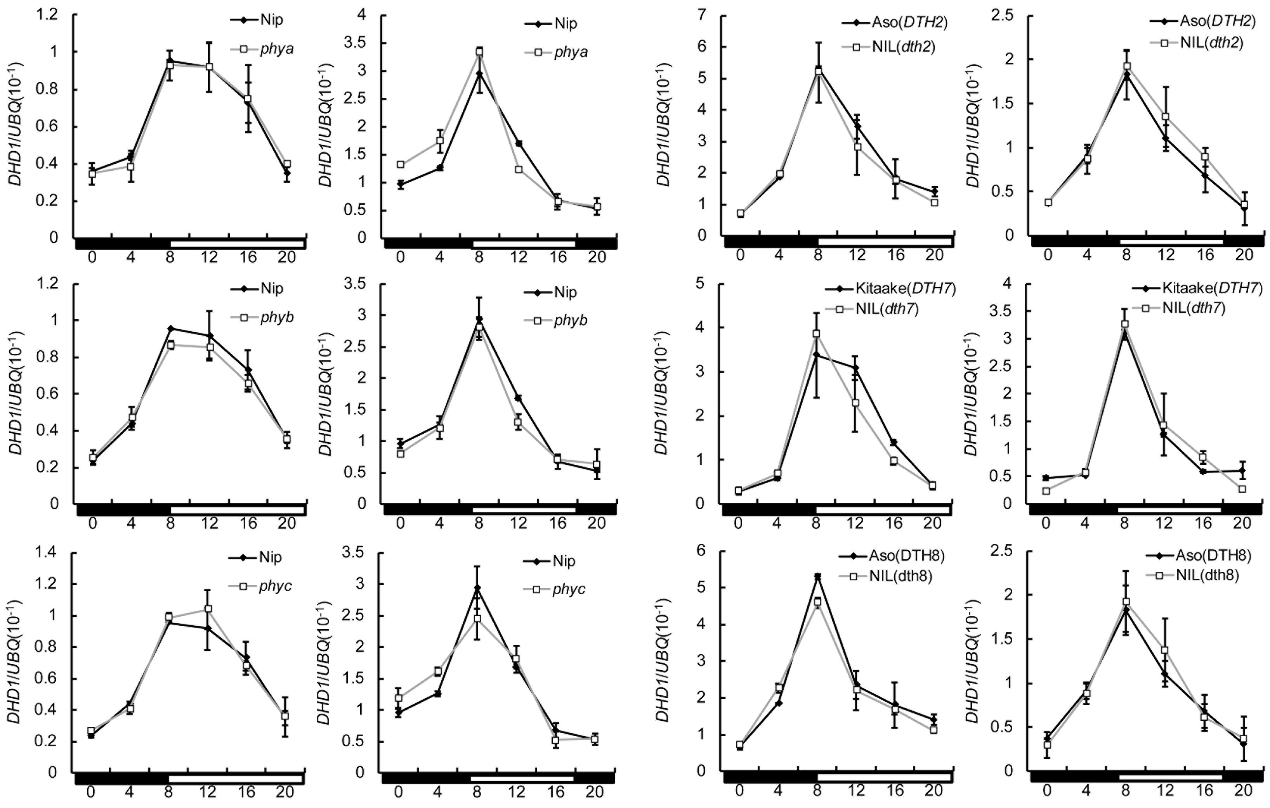


Figure S9. The relative expression levels of *DHD1* in heading date-related mutants or near-isogenic lines. Means ± SE. Black and white boxes denote dark and light periods, respectively.


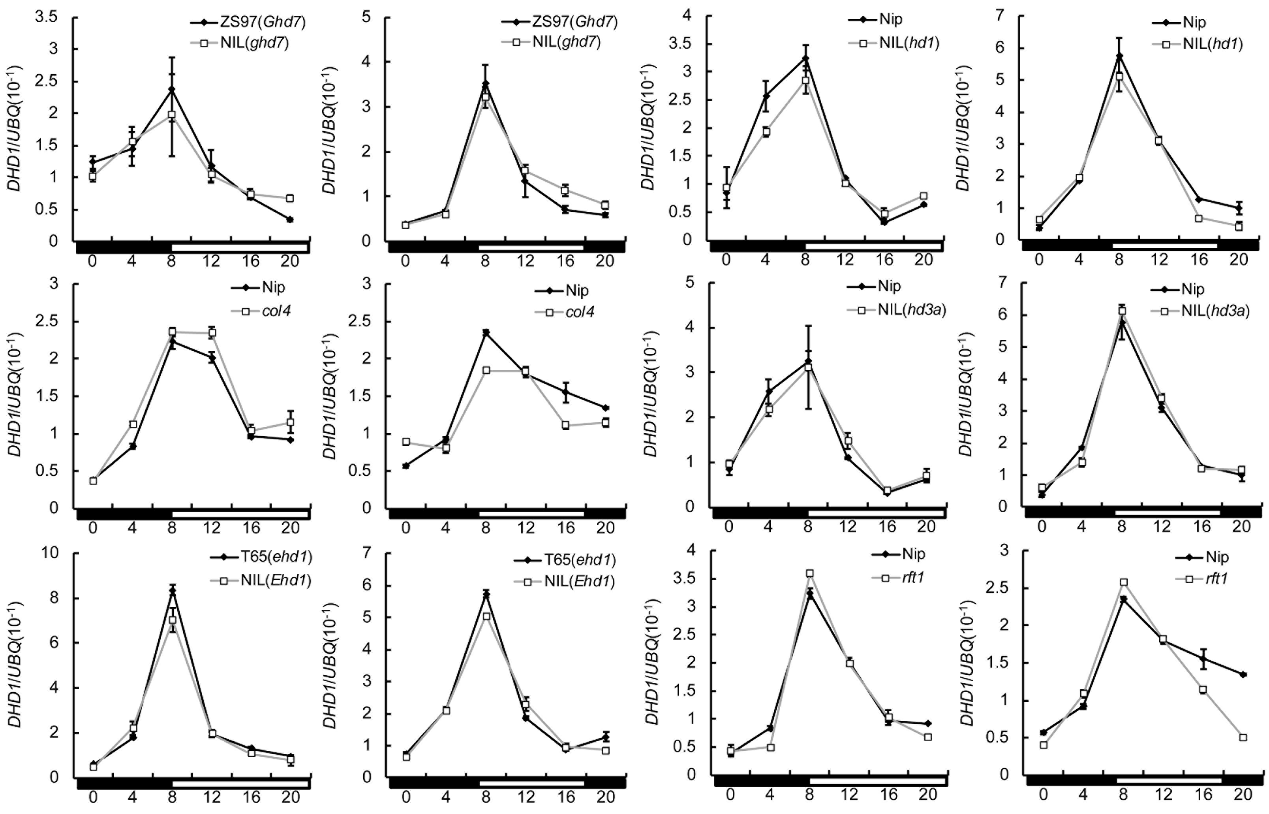


Figure S10. The relative expression levels of *DHD1* in the heading date-related mutants or near-isogenic lines. Means ± SE. Black and white boxes denote dark and light periods, respectively.





Figure S11. Accumulation of DHD1-Flag protein in rice protoplasts subjected to ABA, GA and BR hormone treatments. Rice Heat Shock Protein (HSP) was used as an internal control protein.


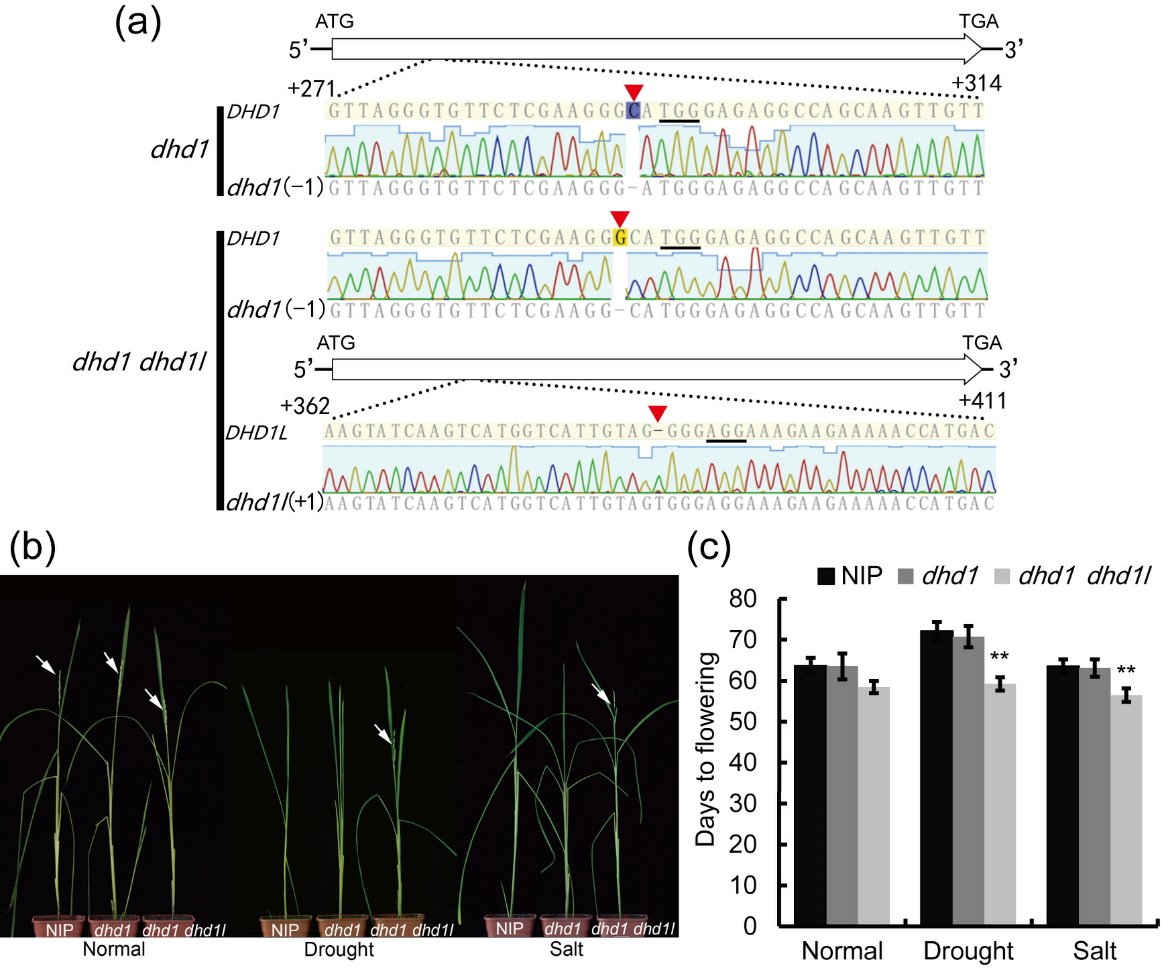


Figure S12. Flowering time of NIP, *dhd1* and *dhd1 dhd1l* under normal, salt and drought conditions in SD climate chamber. (a) Diagram of mutation sites in *dhd1* and *dhd1l dhd1l* mutants. (b) Phenotypes of NIP, *dhd1* and *dhd1 dhd1l* under normal, drought and salt conditions. (c) Flowering time of NIP, *dhd1* and *dhd1 dhd1l* under normal, drought and salt conditions. Means ± SE (n>10), ***p*≤0.01.


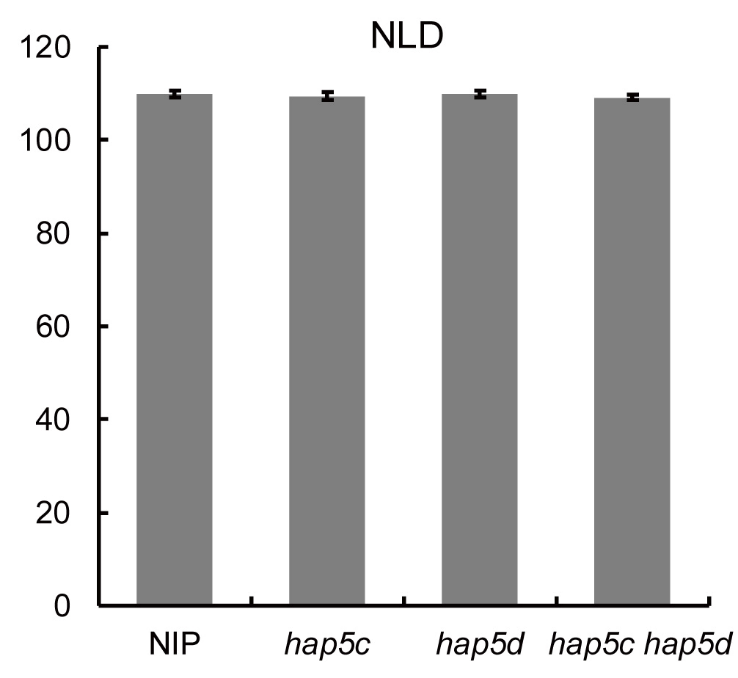


Figure S13. Heading date of *hap5c*, *hap5d* and *hap5c* *hap5d* grown in the field under NLD condition. Means ± SE (*n*>12).
