# Supplementary material for: DELAYED HEADING DATE1 interacts with OsHAP5C/D, delays flowering time and enhances yield in rice
Source: Plant Biotechnol J. 2018 Sep 4;17(2):531–9. doi: 10.1111/pbi.12996 (PMC6335081; doi:10.1111/pbi.12996)
Supplement: Supplementary file 2 — Table S1 Primers used in the current work. [file PBI-17-531-s001.docx]

Table S1. Primers used in the current work.

| Primer name | Sequence |
| --- | --- |
| For vector construction and detection | |
| 1390FLAG-DHD1-F | TCTGCACTAGGTACCTGCAGATGGCCAACCCAGAGGAT |
| 1390FLAG-DHD1-R | ATGGATCCGTCGACCTGCAGTCCGGAGGAAGTGTCTTG |
| 1390FLAG-DHD1L-F | TCTGCACTAGGTACCTGCAGATGGCCAACCCAGAGGATTTC |
| 1390FLAG-DHD1L-R | ATGGATCCGTCGACCTGCAGTCCGGAGGAATTGTCTTGTTG |
| 1390FLAG-HAP1-F | TCTGCACTAGGTACCTGCAGATGGACAACCAGCAGCTACCCTAC |
| 1390FLAG-HAP1-R | ATGGATCCGTCGACCTGCAGTTCGGAGCTTGGAGGTGCAG |
| 1390FLAG-HAP2-F | TCTGCACTAGGTACCTGCAGATGGAGCCCAAATCCACCAC |
| 1390FLAG-HAP2-R | ATGGATCCGTCGACCTGCAGCTGCGGCTGGACATAGTAGTAGG |
| 1390RNAi- DHD1-F1 | CGTAGTCGACGGATCCAGGGACACAGGAGGCTGGTGC |
| 1390RNAi- DHD1-R1 | GAATTCCCGGGGATCCCCGTAAGTCCACCACCTTGC |
| 1390RNAi- DHD1-F2 | TTACTTCTGCACTAGGTACCCCGTAAGTCCACCACCTTGC |
| 1390RNAi- DHD1-R2 | TAGAGCTCAGGCCTGGTACCAGGGACACAGGAGGCTGGTGC |
| DHD1-CRISPR-F | AGATGATCCGTGGCAAGGGTGTTCTCGAAGGGCATGTTTTAGAGCTATGC |
| DHD1-CRISPR-R | GCATAGCTCTAAAACATGCCCTTCGAGAACACCCTTGCCACGGATCATCT |
| DHD1-double-CRISPR-F | AGATGATCCGTGGCAAGGGTGTTCTCGAAGGGCATGTTTTAGAGCTATGC |
| DHD1L-double-CRISPR-R | GCATAGCTCTAAAACCCCCTACAATGACCATGACTCTGAGCCTCAGCGCAGCAGCTTA |
| PAN580- DHD1-F | GCCCAGATCAACTAGTATGGCCAACCCAGAGGATTT |
| PAN580- DHD1-R | TCGAGACGTCTCTAGATCCGGAGGAAGTGTCTTGTG |
| pAN580-HAP5C-F | GCCCAGATCAACTAGTATGGACAACCAGCAGCTACCCTAC |
| pAN580-HAP5C-R | TCGAGACGTCTCTAGATTCGGAGCTTGGAGGTGCAG |
| pAN580-HAP5D-F | GCCCAGATCAACTAGTATGGAGCCCAAATCCACCAC |
| pAN580-HAP5D-R | TCGAGACGTCTCTAGACTGCGGCTGGACATAGTAGTAGG |
| BD- DHD1-F | CATGGAGGCCGAATTCATGGCCAACCCAGAGGATTTC |
| BD- DHD1-R | GCAGGTCGACGGATCCTCATCCGGAGGAAGTGTCTTGTG |
| AD-HAP5C-F | GGAGGCCAGTGAATTCATGGACAACCAGCAGCTACCCTAC |
| AD-HAP5C-R | CGAGCTCGATGGATCCTTCGGAGCTTGGAGGTGCAG |
| AD-HAP5D-F | GGAGGCCAGTGAATTCATGGAGCCCAAATCCACCAC |
| AD-HAP5D-R | CGAGCTCGATGGATCCCTGCGGCTGGACATAGTAGTAGG |
| BIFC- DHD1-F | AGGCCTGGCGCGCCACTAGTGGATCCATGGCCAACCCAGAGGATTT |
| BIFC- DHD1-R | CCGGGAGCGGTACCCTCGAGGTCGACTCCGGAGGAAGTGTCTTGTG |
| BIFC-HAP5C-F | AGGCCTGGCGCGCCACTAGTGGATCCATGGACAACCAGCAGCTACCCTAC |
| BIFC-HAP5C-R | CCGGGAGCGGTACCCTCGAGGTCGACTTCGGAGCTTGGAGGTGCAG |
| BIFC-HAP5D-F | AGGCCTGGCGCGCCACTAGTGGATCCATGGAGCCCAAATCCACCAC |
| BIFC-HAP5D-R | CCGGGAGCGGTACCCTCGAGGTCGACCTGCGGCTGGACATAGTAGTAGG |
| GST- DHD1-F | CTGGTTCCGCGTGGATCCATGGCCAACCCAGAGGATTT |
| GST- DHD1-R | GCGGCCGCTCGAGTCGACTCCGGAGGAAGTGTCTTGTG |
| MBP-HAP5C-F | ATTTCAGAATTCGGATCCATGGACAACCAGCAGCTACCCTAC |
| MBP-HAP5C-R | GCTTGCCTGCAGGTCGACTTCGGAGCTTGGAGGTGCAG |
| MBP-HAP5D-F | ATTTCAGAATTCGGATCCATGGAGCCCAAATCCACCAC |
| MBP-HAP5D-R | GCTTGCCTGCAGGTCGACCTGCGGCTGGACATAGTAGTAGG |
| pAN580-Flag-R | TCGAGACGTCTCTAGAGGATCCTCAGGGCCCCCC |
|  |  |
| For RT-qPCR |  |
| qDHD1-F | GCAGCTGTGCCAATGAGTTTCG |
| qDHD1-R | AACTGTCTCCCACTTTGCTGCTG |
| UBQ-F | ACCACTTCGACCGCCACTACT |
| UBQ-R | ACGCCTAAGCCTGCTGGTT |
| qGI-F | ATCGTTCTGCAGGCCGAGA |
| qGI-R | TCACCAATGCTTCTGGGCTAT |
| qHd1-F | CGTTTCGCCAAGAGATCAG |
| qHd1-R | AGATAGAGCTGCAGTGGAGAAC |
| qEhd1-F | AACCCGGTCATCCTCCAT |
| qEhd1-R | TCATCTCTCACCTCATTTTCT |
| qEhd2-F | CGACGACAATAGCTCGATCGC |
| qEhd2-R | GTGCATGGTCACGGAGCCTT |
| qEhd3-F | GACCACCTCGTCACCTACAAG |
| qEhd3-R | GAGTGTCCCTCCAGCTAATCC |
| qEhd4-F | CAGCCAGCGGAATCATCAC |
| qEhd4-R | CCAAATCCATCAGACCTACTCCT |
| qMADS50-F | GACCGTAACATCAACACCAC |
| qMADS50-R | GAGATCCAGCTTATTCCTGG |
| qMADS51-F | GTTTGCTCTGCTCCTACTC |
| qMADS51-R | ACTCCTCCTCCAGCATTGAA |
| qHd3a-F | GCTAACGATGATCCCGAT |
| qHd3a-R | CCTGCAATGTATAGCATGC |
| qMADS14-F | CAACCTCAAACAAGTTCCTC |
| qMADS14-R | TGCTGCTACATCCTCTATCC |
| qDTH8-F | CAGGAGTGCGTGTCGGAGTT |
| qDTH8-R | GGTCGTCGCCGTTGATGGT |
| qMADS56-F | AAGAGCCTCCACAAGATAAGA |
| qMADS56-R | TCGTCGTCATGTGGTTAGC |
| qPHYA-F | AGCCTGACCAAGAACAGCATTGG |
| qPHYA-R | TGCCCTGGTGCTTGATCCTAAG |
| qPHYB-F | CTCATCTTCAAGGAATCTGAGG |
| qPHYB-R | CCTGCTAGAACAAGCATTCAC |
| qPHYC-F | AAGGGCCTATTGTGCTCCAAGTC |
| qPHYC-R | AGCTGGATGGACAAGCCTGAAC |
| qDTH2-F | CCAGTTTCAACGACGCCTAA |
| qDTH2-R | GTCTCCATATACGCTCCCATCA |
| qSE5-F | AGGACTCCCAAGCTTTTATC |
| qSE5-R | CTCCAGAATACGAGAACGAC |
| qCOL4-F | GTCCATGGACGGAATCAAGG |
| qCOL4-R | CTCCGACGACGACAAGCTGT |
| qRFT1-F | TGACCTAGATTCAAAGTCTAATCCTT |
| qRFT1-R | TGCCGGCCATGTCAAATTAATAAC |
